# A systematic review and meta-analysis of the use of packing in the management of perianal abscesses

**DOI:** 10.1308/rcsann.2023.0108

**Published:** 2024-04-02

**Authors:** DL Crook, OME Padfield

**Affiliations:** The Royal London Hospital, UK

**Keywords:** Perianal abscesses, Abscess packing, Recurrence

## Abstract

**Background:**

Perianal abscesses are common presentations and reasons for emergency general surgery admissions. Management involves incision and drainage of the abscess and packing the cavity with internal wound dressings. This meta-analysis aimed to assess in adults if packing an abscess or leaving it unpacked leads to a significant difference in the outcomes of pain on wound dressing, time to healing, rate of fistulation and abscess recurrence.

**Methods:**

Randomised controlled trials (RCTs) with participants aged 18 years or older that compared packing of perianal abscess cavities with no packing between 2002 and 2022 were searched for in December 2022 on OVID Medline and Embase, the CENTRAL register of trials, PubMed and Google Scholar. Risk of bias was assessed using the Cochrane Risk of Bias tool. Random effects meta-analysis was conducted on the data extracted.

**Results:**

Three RCTs involving 490 patients were analysed for the outcomes of abscess recurrence and postoperative fistula formation; the data were not adequate to assess pain on dressing and time to healing. For unpacked versus packed, the pooled relative risk of abscess recurrence was 1.57 (95% confidence interval (CI) 0.764, 3.29, *p*=0.219) and for fistula formation 0.686 (95% CI 0.430, 1.09, *p*=0.114). These results suggest there is no significant benefit to packing abscess cavities.

**Conclusions:**

Analysis of the outcomes suggests there is no significant difference with regards to rates of abscess recurrence or fistula formation between the packed and unpacked groups; however, appropriately powered RCTs are required in this area to provide more primary evidence to inform best practice and clinical management.

## Introduction

A perianal abscess is a subcutaneous or submucosal superficial collection of pus located in the perianal region overlying the intersphincteric space. Deeper anorectal abscess can be further classified according to anatomical location or direction of extension, such as intersphincteric (between internal and external anal sphincter), supralevator (superiorly to the pelvic floor), ischiorectal (an abscess that extends into the ischiorectal fossa beneath the levator ani) or horseshoe (crossing the midline).^1^

The aetiology of perianal and anorectal sepsis is mostly of cryptoglandular origin from infected or obstructed anal glands.^2^ It is a common general surgical presentation, with incidence reported to be 20.2 to 40 per 100,000 per year in the UK.^3^ Patients report symptoms of perianal pain, swelling or lump, fever and purulent discharge, and have evidence of leucocytosis on blood tests. Risk factors for developing perianal abscess include nonmodifiable factors such as inflammatory bowel disease, diabetes, being in the fourth decade of life and male sex (male:female 2:1) and modifiable factors such as smoking.^4^

Standard surgical management of perianal abscess involves external incision and drainage procedure, usually under general anaesthetic to enable adequate examination and management. Historically, the incised wound is left open to heal by secondary intention, with the remaining abscess cavity packed with internal wound dressings, such as ribbon gauze or alginate dressings. Changing and continued repacking of the wound requires regular ongoing community nursing input throughout the postoperative period until healed. Patient feedback reports this postoperative wound care is painful and therefore impacts on patient experience.^5^ This is clinically taught to be to prevent abscess recurrence and to allow the cavity to continue to drain; however, there is little to no scientific evidence to support this practice.^6^

There are currently no UK national clinical evidence-based guidelines on which to base this ongoing surgical practice, and it is not recommended by the Association of Coloproctologists of Great Britain and Northern Ireland, or clinical guidelines in the US or Germany.^7–9^ There have been recent randomised controlled trials (RCTs) conducted to assess whether there is any clinical benefit for patients undergoing postoperative packing of perianal abscesses, whose outcomes we have meta-analysed.

## Methods

This study was registered prospectively with PROSPERO before data collection began (registration ID CRD42022372977) and has been reported in accordance with the PRISMA 2020 statement.^10^ A systematic review of Ovid Medline, Ovid EMBASE, The Cochrane Central Register of Controlled Trials (CENTRAL), PubMed and Google Scholar was conducted on 2 December 2022 for relevant studies published between 1 January 2002 and 2 December 2022.

The search terms used were perianal OR abscess, perianal AND abscess, perianal abscess, perirectal OR abscess, perirectal AND abscess, perirectal abscess, perirectal abscess AND packing, perianal abscess AND packing; results were restricted to return only those published between 2002 and 2022. Studies were excluded if they were not published in English, if they had been published more than 20 years ago, were not RCTs or included participants younger than 18 years of age. Abstracts published as the result of conference proceedings, dissertations and other grey literature were not included.

**Figure 1:**
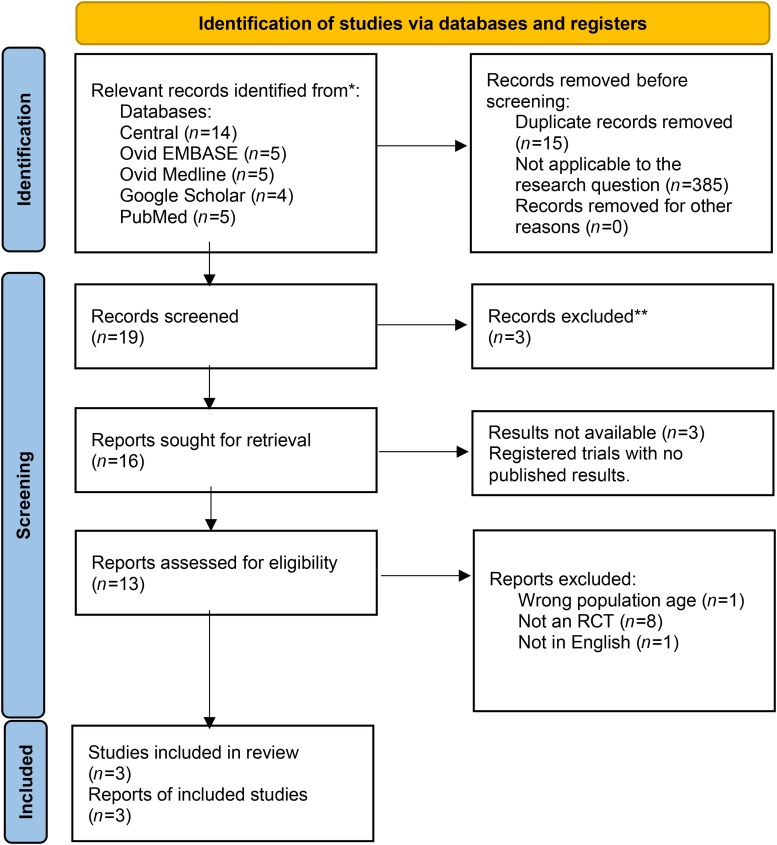
Diagram of the flow of trials through the study, from the template published by PRISMA^10^ RCT = randomised controlled trial.

Nineteen relevant records were returned, of which three met the inclusion criteria; the studies by Newton, Perera and Tonkin.^5,11,12^ The flow of trial records is shown in Figure 1. Data were extracted by both investigators independently. Data on time to healing and pain on dressing were not comparable due to reporting differences and so only data on recurrence and fistula were analysed. Risk of bias assessments were carried out on each of the included studies using the Cochrane Risk of Bias 2 (RoB2) tool, again by both investigators independently. The characteristics of the included studies are listed in Table 1. Data extracted on recurrence and fistula formation are shown in Table 2 and Table 3, respectively. GRADE assessments of the quality of the evidence were carried out on each paper and the results of these are presented in Table 4. A summary of findings table formed of the findings of the RoB2, GRADE assessment and the meta-analysis is shown in Table 5.

**Table 1:** Characteristics of included studies

Study name	Year of publication	Number recruited	Study type	Randomisation method	Setting	Country
Postoperative Packing of Perianal Abscess Cavities (PPAC2): randomized clinical trial, Newton 2022^5^	2022	433	Multicentre RCT	1:1 using an online tool (the Treatment Allocation RanDomIsation System (TARDIS))	Acute secondary and tertiary care hospitals	UK
A pilot randomised controlled trial evaluating postoperative packing of the perianal abscess, Perera 2015^11^	2015	14	Single centre, pilot RCT	Sealed, numbered opaque envelopes	An acute tertiary care hospital	UK
Perianal abscess: a pilot study comparing packing with nonpacking of the abscess cavity, Tonkin 2004^12^	2004	43	Single centre, pilot RCT	Sealed, numbered opaque envelopes	An acute tertiary care hospital	Australia

**Table 2:** Data extracted on abscess recurrence

Study	Packed recurred	Packed not recurred	Not packed recurred	Not packed not recurred
Newton, 2022	7	206	13	210
Perera, 2015	3	5	2	4
Tonkin, 2004	1	19	3	20

**Table 3:** Data extracted on fistula formation

Study	Packed fistula	Packed no fistula	Not packed fistula	Not packed no fistula
Newton, 2022	32	181	24	196
Perera, 2015	1	7	0	6
Tonkin, 2004	4	16	2	21

**Table 4:** GRADE assessment of the evidence from the included RCTs

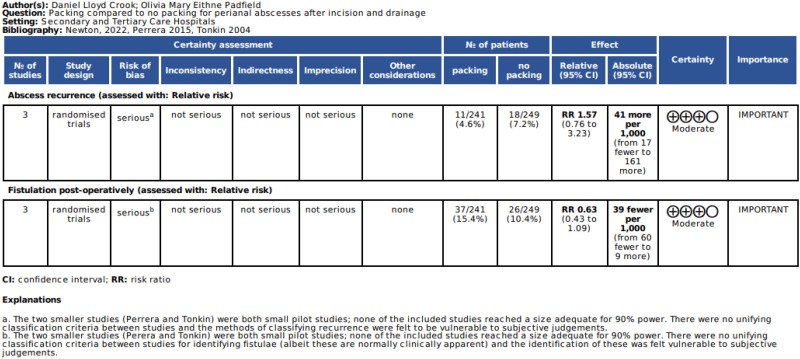

**Table 5:** Summary of findings table

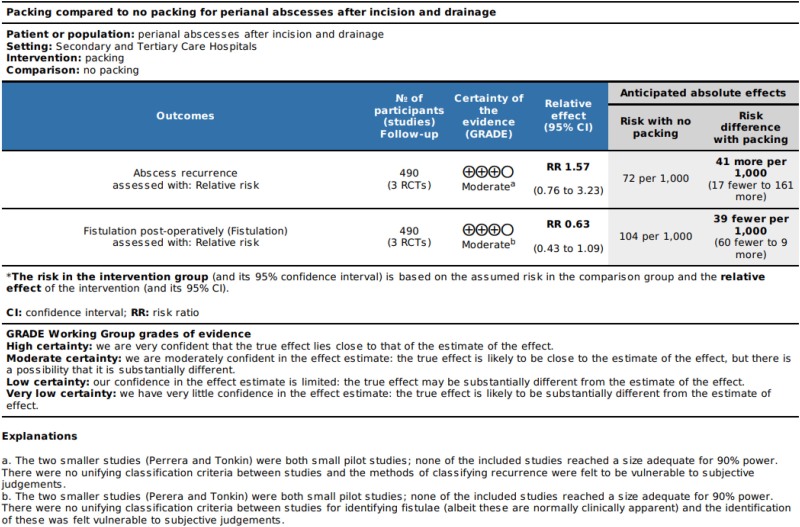

Data extracted were processed using R 4.0.1, ‘Shake and Throw’. Using the meta package, standard DerSimonian and Laird random effects meta-analysis was performed due to the heterogeneity of the included studies and the likelihood that there is no one true effect size for the outcomes measured. Hartung – Knapp adjustments were not used in the modelling. The relative risk (RR) was used as the summary measure for the model output. As a sensitivity analysis, fixed effects meta-analysis was also performed, which produced results similar to those of the random effects meta-analysis.

Intended subgroup analyses as investigations of sources of heterogeneity were not conducted due to the limited data available in the studies on the subgroups it was intended to investigate. Funnel plots of standard error versus effect size were not generated due to the small number of studies included in the analysis. *I*^2^ was the chosen measure of heterogeneity and a prespecified alpha of 0.05 was used for significance.

## Results

Meta-analyses were performed on the data gathered on abscess recurrence and fistula formation, as planned and detailed in the PROSPERO registration. There were 490 patients in the recurrence meta-analysis and 29 recurrent abscesses were recorded, giving an overall recurrence rate of 5.91%. By packed versus not packed, the respective percentages were 4.56% and 7.93%. The pooled relative risk of recurrence for patients without packing was 1.57 (95% confidence interval (CI) 0.764, 3.29) and the *p* value was 0.219, showing an insignificant difference between the randomised groups.

**Figure 2:**
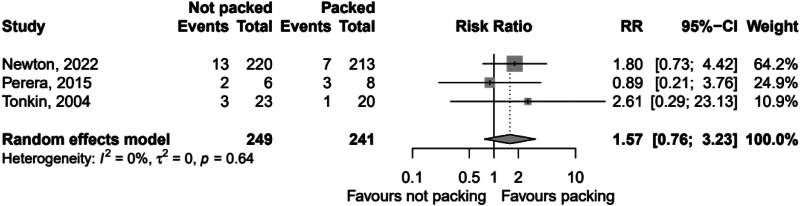
Forest plot of pooled RR of abscess recurrence with and without packing CI = confidence interval; RR = relative risk.

**Figure 3:**
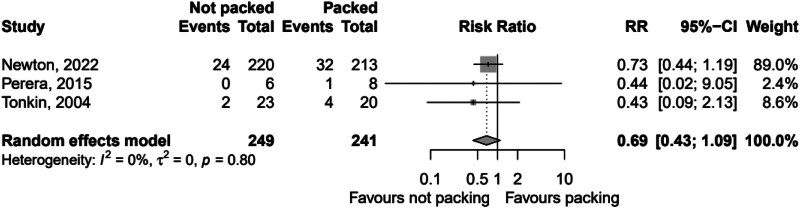
Forest plot of pooled RR of fistula formation with and without packing CI = confidence interval; RR = relative risk.

**Figure 4:**
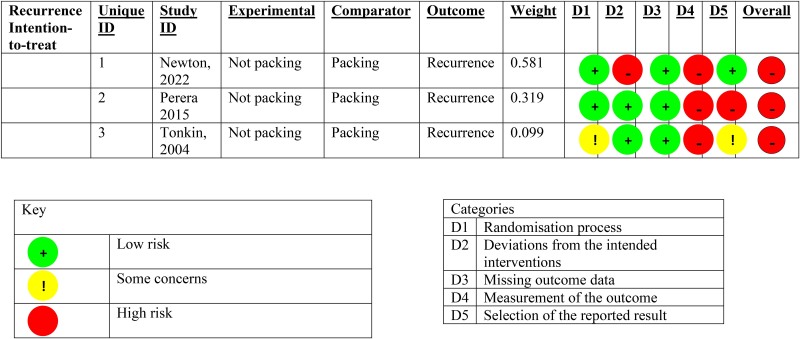
Summary diagram of the risk of bias in the trials for the outcome of recurrence, from the Cochrane RoB2 tool

**Figure 5:**
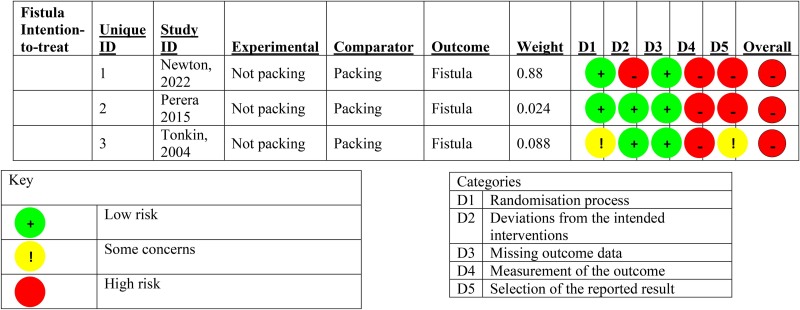
Summary diagram of the risk of bias in the trials for the outcome of fistula formation, from the Cochrane RoB2 tool

There was insignificant heterogeneity detected between the studies (*I*^2^ 0.0% (95% CI 0.0, 89.6%, *p*=0.640). The second analysis comprised 490 patients with 63 recorded fistulae, giving an overall fistulation rate of 12.9%. By packed versus not packed, the respective fistulation rates were 15.4% and 10.4%. The pooled relative risk of fistulation without packing was 0.686 (95% CI 0.430, 1.09), *p*=0.114, showing a nonstatistically significant difference in fistulation. Heterogeneity between studies was insignificant, with *I*^2^ 0.0% (95% CI 0.0, 89.6%), *p*=0.800. The forest plots for these analyses are Figures 2 and 3 respectively. The studies for both recurrence and fistulation were found to be at high risk of bias (Figures 4 and 5). The GRADE assessment found there to be moderate confidence in the evidence. Data are available on fair application to the authors.

## Discussion

These data show no significant differences for the outcomes of abscess recurrence and fistulation whether or not a perianal abscess is left with packing. The lack of superiority of packing for preventing recurrence and fistulation supports leaving abscesses unpacked and instead simply dressed. This, however, is based on evidence assessed as being of mostly poor quality with doubts particularly over the methods used to classify the outcomes, with no explicit criteria in the papers to demonstrate how the conclusions of recurrence or fistula were reached. Follow-up and dropout was a significant issue with Newton, as around half of the patients completed the trial per protocol (229/433, 52.9% completion rate).

Although this rate is grossly comparable across both arms with a similar dropout rate in each, there is no analysis of this rate by patient demographics, making analysis of whether there were differential dropout rates by patient group difficult. Unfortunately, the data were not adequate to allow comparison of the outcomes of pain on dressing and time to healing between the studies.

The papers are all underpowered to detect a true difference in the groups they investigate with regard to fistulation and recurrence, although Perera and Tonkin were both published as pilot studies and the latter acknowledged the limitations of their resources in determining the number recruited. For an alpha of 0.05 and power of 90%, retrospective power calculations using the pwr package in R show that, for the pooled incidence detected across the studies, a sample size of 1,065 per group would be required to detect a true difference between the groups for the outcome of recurrence and 937 per group for the outcome of fistulation. These would give total numbers needed as 2,130 and 1,874, respectively, without allowing for any drop out or loss to follow-up.

None of the included studies reached these numbers of participants. There was a disparity in the number of participants in the studies included, with the paper by Newton the largest. This raised consideration of small study effects and overestimation of the treatment effect in Perrera and Tonkin; however, small study effects were not observed in the outcomes included in this analysis so no adjustment was made for study size.

There is a large health economics benefit in not packing an abscess postoperatively. Time and healthcare costs are saved by reducing the need for repeated visits to have dressings changed, with a concomitant reduction in the burden of work placed on community services, which are under severe pressures. Patients are able to resume work and obtain a more normal existence if they do not need to attend multiple dressings appointments, aiding their physical and socioeconomic recovery. Environmental benefits are also accrued through not packing perianal abscesses by minimising carbon emissions from patient travel and reducing the waste from packaging materials. Minor reductions in time spent in theatre and volume of highly polluting anaesthetic gases used per operation can also be expected.

The evidence from this meta-analysis suggests that there may be no significant benefit in the use of packing in perianal abscess wounds after incision and drainage. The recommendations of this meta-analysis are not strong, as they are based on generally weak evidence. Pairwise comparisons for the outcomes of time to healing and pain on wound dressing were not possible, and whether there is a significant difference between packing and no packing for these outcomes is not established by a recent meta-analysis. Adequately powered RCTs, with objective criteria published for addressing outcomes and suitable blinding of assessors, are required to investigate whether to pack perianal abscesses after incision and drainage.

Given the acute nature of presentations with perianal abscesses and the short time lag between admission and theatre, the coordination of consent and recruitment for large multicentre trials in this area would be troublesome. Further, maintaining a high follow-up rate for repeated personal examination may be difficult due to both patient and service-level factors, and the difficulty in maintaining high follow-up rates is reflected in the number who dropped out of the study by Newton. Although this is an area meriting further investigation, the studies required have the potential to not be straightforward to conduct.
